# Supplementary material for: Structural basis of protein translocation by the Vps4-Vta1 AAA ATPase
Source: eLife. 2017 Apr 5;6:e24487. doi: 10.7554/eLife.24487 (PMC5413351; doi:10.7554/eLife.24487)
Supplement: Supplementary file 1. — DOI: http://dx.doi.org/10.7554/eLife.24487.032 [file elife-24487-supp1.docx]

**Supplementary File 1**

**Proteins and Expression Vectors**

| **Protein** | **Plasmid Name** | **Internal ID** | **Addgene ID** | **Notes** |
| --- | --- | --- | --- | --- |
| Vps4 full-length | pET151-Vps4 | CPH3391 | 87733 | [1] |
| Vps4^81-437^ | pET151-Vps4(81-437) | CPH3392 | 87734 | [2] |
| Vps4^101-437^ | pET151-Vps4(101-437) | CPH3393 | 87735 | [3] |
| Vps4^101-437^ L151D | pET151-Vps4(101-437,L151D) | CPH3084 | 87736 | [4] |
| Vps4^101-437^-Hcp1 | pET151-Vps4(101-437)-Hcp1 | CPH3188 | 87737 | [5] |
| Vps4^101-437^-Hcp1 K321A | pET151-Vps4(101-437)-Hcp1 K321A | CPH3527 | 87747 | [6] |
| Vps4^101-437^-Hcp1 E322A | pET151-Vps4(101-437)-Hcp1 E322A | CPH3528 | 87748 | [6] |
| Vps4^101-437^-Hcp1 R325D | pET151-Vps4(101-437)-Hcp1 R325D | CPH3560 | 89289 | [6] |
| Vta1^VSL^ | pET151-Vta1(280-330) | CPH3388 | 87738 | [7] |
| Vta1^VSL^ S278C | pET151-Vta1(278-330, S278C) | CPH3389 | 87739 | [7] |

[1] Modified from pET151-D-Topo-Vps4p (Monroe et al., 2014) to remove vector-encoded sequence between TEV cleavage site and insert

[2] Modified from pET151-D-Topo-Vps4p(81-437) (Han et al., 2015) to remove vector-encoded sequence between TEV cleavage site and insert

[3] Generated from pET151-Vps4

[4] Generated by site-directed mutagenesis of pET151-Vps4(101-437)

[5] *P. aeruginosa* Hcp1 was amplified from pDONR201-Hcp1 (DNASU clone ID PaCD00006037) and inserted into pET151-Vps4(101-437)

[6] Generated by site-directed mutagenesis of pET151-Vps4(101-437)-Hcp1

[7] Modified from pET151-D-Topo-Vta1p(280-330) (Han et al., 2015) to remove vector-encoded sequence between TEV cleavage site and insert
